# A Rare Case of Folate Deficiency-Induced Subacute Combined Degeneration of the Spinal Cord: A Case Report

**DOI:** 10.7759/cureus.42593

**Published:** 2023-07-28

**Authors:** Lohitha Guntupalli, Andrew Lurie, Veenah Stoll, Luis Daniel Lugo

**Affiliations:** 1 Osteopathic Medicine, Nova Southeastern University Dr. Kiran C. Patel College of Osteopathic Medicine, Clearwater, USA; 2 Internal Medicine, Nova Southeastern University Dr. Kiran C. Patel College of Osteopathic Medicine, Clearwater, USA; 3 Basic Sciences, Nova Southeastern University Dr. Kiran C. Patel College of Osteopathic Medicine, Clearwater, USA; 4 Medical Education, Lakeland Regional Health, Lakeland, USA

**Keywords:** spastic paresis, proprioception, vit b12 deficiency, folate deficiency, subacute combined degeneration

## Abstract

Folate deficiency is a rare cause of subacute combined degeneration (SCD). SCD is characterized by demyelination of the dorsal and lateral columns of the cervical and thoracic spine most commonly caused by vitamin B12 deficiency. Folate deficiency, which can be clinically indistinguishable from B12 deficiency, leads to a variety of presentations, including absent vibratory sensation and proprioception, sensory ataxia, and progressive motor weakness. Recognizing the clinical sequelae of SCD is essential for timely diagnosis and treatment. We present a case of a 38-year-old male with progressive ascending bilateral lower extremity numbness and motor weakness in the presence of normal vitamin B12 and methylmalonic acid levels. MRI of the entire neuraxis revealed a diagnosis of SCD, which was managed with oral folate replacement. We aim to highlight this rare cause of SCD, which can lead to permanent neurological deficits if not promptly recognized and treated.

## Introduction

Subacute combined degeneration (SCD) is a neurological degeneration that results from demyelination of the dorsal and lateral columns of the spinal cord. Clinical findings include symmetrical impaired tactile discrimination, proprioception, and vibration sensation as a result of damage to the dorsal columns. Lateral column degeneration often presents as muscle weakness, stiffness, and diffuse hyperreflexia. Patients may also have additional signs and symptoms of peripheral nerve involvement, visual deficits, and neuropsychiatric disease if SCD remains untreated. Diagnosis is made on MRI of the spinal column, which reveals demyelination observed as bilateral paired regions of T2 hyperintensity in the dorsal or lateral columns on the cervical and/or thoracic spinal cord [1].

SCD is most commonly acquired due to vitamin B12 deficiency that results from an abnormality in its absorption through mechanisms such as inadequate intake, pernicious anemia, or chronic gastritis. Vitamin B12 plays an integral role in DNA synthesis and fatty chain metabolism, which are required for neuronal myelin synthesis and maintenance. Decreased levels contribute to the interruption of methylmalonyl-CoA mutase, which is an important enzyme necessary for myelin synthesis [2-4]. In rare cases, clinical vitamin B12 deficiency can persist despite normal measured levels. In these instances, methylmalonic acid accumulation, which has a much higher sensitivity for vitamin B12 levels, can provide some insight [5].

In patients presenting with absent vibratory sensation and proprioception, sensory ataxia, and progressive motor weakness, laboratory workup should include folate deficiency, copper deficiency, zinc excess, and pertinent infectious diseases. Folate deficiency is often clinically indistinguishable from vitamin B12 deficiency, leading to delayed time to treatment [2,6]. As such, clinical suspicion for additional causes of SCD, despite normal levels of vitamin B12 and methylmalonic acid, should remain high.

## Case presentation

A 38-year-old Caucasian male, with a past medical history of coronary artery disease, myocardial infarction, and hyperlipidemia who originally presented to the emergency department complaining of refractory abdominal pain, was found to have a six-month history of ascending numbness, motor weakness, and blurry vision. These neurologic symptoms had originated in the toes, followed by progressively bilateral leg weakness and near-complete numbness that rendered him wheelchair-bound for the past two months. The patient was being treated with gabapentin without any symptom alleviation.

On examination, the patient was alert and oriented to person, place, time, and situation. Cranial nerves 2-12 were intact. Strength in the bilateral upper extremities was 4/5, with weak hand grip bilaterally. Strength in the bilateral lower extremities was 3/5. Further neurologic examination showed a loss of vibratory sensation in the bilateral feet with a sensory level of T6. His coordination of the upper extremities remained intact but could not be assessed in the lower extremities due to weakness.

**Figure 1:**
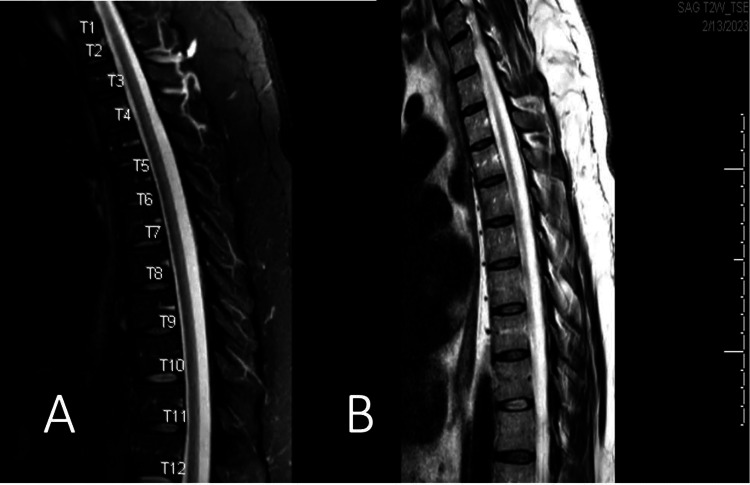
MRI of the thoracic spine without and with contrast revealed T2 hyperintensity of the dorsal columns from T3 to T11, most prominent at T7, without enhancing lesions. A. T1-weighted image B. T2-weighted image

The patient was admitted to the hospital for a more extensive diagnostic workup. Pertinent labs included decreased hemoglobin and severely decreased folate with normal vitamin B12, methylmalonic acid, and copper levels (Table 1). MRI of the thoracic spine without and with contrast revealed T2 hyperintensity of the dorsal columns from T3 to T11, most prominent at T7, without enhancing lesions (Figure 1). The clinical signs and symptoms with investigative findings suggested a diagnosis of SCD secondary to folate deficiency. Treatment was initiated with oral folate, and the patient’s serum levels returned to normal range after one week of repletion therapy. Despite adequate folate replacement, the patient’s neurologic condition and wheelchair-bound state were without appreciable improvement.

**Table 1:** Serum and urine laboratory studies significant for normocytic anemia in the setting of severely low folate. MCV: Mean Corpuscular Volume, VDRL: Venereal Disease Research Laboratory, HIV: Human Immunodeficiency Virus

	Value	Reference Range
Hemoglobin	10.9 g/dL	12-15 g/dL
MCV	96 fL	80-100 fL
Vitamin B12	343 pg/mL	180-914 pg/mL
Folate	<2 ng/mL	>5.9 ng/mL
Methylmalonic Acid	95 nmol/L	87-318 nmol/L
Copper (serum)	82 ug/dL	63.7-140.12 ug/dL
Copper (urine)	25 ug/dL	20-50 ug/dL
Antinuclear antibodies	Negative	Negative
VDRL	Negative	Negative
HIV	Negative	Negative
Aquaporin-4 antibodies	Negative	Negative

Further workup, including lumbar puncture, VDRL, ANA, and AQP-4 antibodies, were all unremarkable (Table 2). More than five oligoclonal bands were found equally in both serum and CSF, indicating a systemic synthesis of gammaglobulins as opposed to intracerebral synthesis. Over the course of this patient’s hospitalization, he continued to complain of progressive bilateral upper extremity weakness despite no appreciable changes on imaging. Infectious etiology and systemic autoimmune diseases were ruled out as potential causes for these worsening complaints. Due to the patient’s delay in seeking medical attention upon symptom initiation, his neurological deficits were likely permanent by the time his folate levels were replenished. The patient's initial complaint of refractory abdominal pain and persistent complaints of nausea and vomiting was evaluated with EGD and showed large amounts of residual food and gastric juice in the stomach. Biopsy confirmed gastritis, which may provide some indication of the etiology of the patient's severe folate deficiency. Repeat CT of the abdomen following Flagyl and IV Rocephin therapy showed some initial improvement. Following a complicated clinical course, the patient was discharged with home hospice.

**Table 2:** Cerebrospinal fluid laboratory studies without appreciable abnormalities. WBCs: White Blood Cells

	Value	Reference Range
WBCs	None	<5 cells
Protein	21 mg/dL	15-45 mg/dL
Glucose	58 mg/dL	50-80 mg/dL

## Discussion

SCD has been reported since the late 1800s, most notably in relation to B12 deficiency secondary to pernicious anemia [7]. SCD is a rare disease whose incidence is poorly studied, likely in part due to the most common cause, pernicious anemia, having an incidence of 0.1% in the general population [8]. Rarely, folate deficiency has been described to present with a similar pathology; however, this is far less common in the United States due to dietary fortification [9-10]. As seen in this patient, folate deficiency can lead to a cluster of symptoms seemingly indistinguishable from the common findings of B12 deficiency, including absent vibratory sensation and proprioception, sensory ataxia, and progressive motor weakness [11-14].

The pathomechanism by which folate deficiency creates neuronal damage is not fully elucidated. Folate functions, along with vitamin B12, as a one-carbon methyl donor. The lack of these methylation reactions reduces the synthesis of nucleotides and may affect other important regulators of neuronal function such as myelin basic protein [2-4]. In cases where there is a high index of suspicion for SCD, folate, copper, and zinc levels should be checked alongside vitamin B12. Although both vitamin B12 and folate deficiencies classically present with macrocytic anemia, a lack thereof cannot solely exclude either diagnosis [15]. Furthermore, some patients may present with a clinical vitamin B12 deficiency despite normal levels seen on laboratory workups. In these cases, concurrent vitamin B12 supplementation, in addition to reversal of the causative etiology, should be considered.

The differential diagnosis of SCD is broad and highly dependent on patient risk factors and clinical presentation. Important diagnoses to consider include copper deficiency, zinc toxicity, transverse myelitis, multiple sclerosis, epidural abscess, and Guillain-Barré syndrome. Accordingly, a proper workup includes measurement of serum and urine copper, serum zinc, and a contrast-enhanced MRI. Imaging findings revealing and contrast-enhancing lesion strongly encourages an alternative diagnosis.

SCD is diagnosed with MRI findings of bilateral paired regions of T2 hyperintensity in the dorsal and lateral columns in the cervical and/or thoracic spinal cord [1]. In this patient with symptoms significant for SCD and confirmatory MRI, a high index of suspicion for folate deficiency should exist. Unlike B12 deficiency, folate deficiency has been shown to delay neurologic return of function after vitamin replacement [2,6]. The patient's CT findings of diffuse colitis may provide some indication of the etiology of his severe folate deficiency-induced SCD. This may also provide an explanation for possible subclinical vitamin b12 deficiency, which could have contributed to the patient's inability to regain neurological function. However, given that patient's unremarkable methylmalonic acid levels and delay in seeking medical attention, this is unlikely. Nevertheless, patients who present with SCD with normal vitamin B12 levels should be considered for folate deficiency and concurrently treated for subclinical vitamin B12 deficiency.

Prompt diagnosis and treatment are imperative as early recognition can lead to the reversal of symptomatology. If not corrected in a timely fashion, deficiency may result in permanent effects like those seen with the patient case described. Consistent with our patient, clinical response time to folate replacement has been reported to be delayed due to a lack of knowledge of folate deficiency as a contributor to SCD. Furthermore, subclinical vitamin B12 deficiency can often obscure a prompt diagnosis of SCD and contribute to an increased delay in neurological return of function after folate replacement [2,6]. In any case, given the prevalence of subclinical B12 deficiency and ambiguity in clinical presentation between folate and B12 deficiency-induced SCD, routine supplementation with both folate and vitamin B12 may be beneficial and lead to a higher likelihood of symptomatology reversal.

## Conclusions

Folate deficiency has become increasingly rare in developed countries, such as the United States, that fortify foods. Nevertheless, both vitamin B12 and folate levels should be evaluated in a patient presenting with symptoms of ascending motor weakness, absent vibratory sensation and proprioception, and blurry vision. Clinical suspicion for folate deficiency and subclinical B12 deficiency contributing to SCD in the presence of normal vitamin B12 and methylmalonic acid levels should remain high. Despite similar symptoms, folate deficiency, unlike B12 deficiency, has been shown to have a delay in neurologic return of function after vitamin replacement. Thus, prompt diagnosis and treatment with supplementation of both B12 and folate are essential to minimize permanent defects.
